# Short-term, medium-term, and long-term risks of nonvariceal upper gastrointestinal bleeding after dengue virus infection

**DOI:** 10.1371/journal.pntd.0010039

**Published:** 2022-01-19

**Authors:** Yu-Wen Chien, Hui-Ning Chuang, Yu-Ping Wang, Guey Chuen Perng, Chia-Yu Chi, Hsin-I Shih

**Affiliations:** 1 Department of Public Health, College of Medicine, National Cheng Kung University, Tainan, Taiwan; 2 Department of Occupational and Environmental Medicine, National Cheng Kung University Hospital, College of Medicine, National Cheng Kung University, Tainan, Taiwan; 3 Institute of Basic Medical Sciences, College of Medicine, National Cheng Kung University, Tainan, Taiwan; 4 Department of Microbiology and Immunology, College of Medicine, National Cheng Kung University, Tainan, Taiwan; 5 Center of Infectious Disease and Signaling Research, National Cheng Kung University, Tainan, Taiwan; 6 National Mosquito-Borne Diseases Control Research Center, National Health Research Institutes, Miaoli, Taiwan; 7 Department of Emergency Medicine, National Cheng Kung University Hospital, College of Medicine, National Cheng Kung University, Tainan, Taiwan; 8 School of Medicine, College of Medicine, National Cheng Kung University, Tainan, Taiwan; Fundação Oswaldo Cruz: Fundacao Oswaldo Cruz, BRAZIL

## Abstract

Dengue patients have an increased risk of acute gastrointestinal (GI) bleeding. However, whether dengue virus (DENV) infection can cause an increased long-term risk of GI bleeding remains unknown, especially among elderly individuals who commonly take antithrombotic drugs. A retrospective population-based cohort study was conducted by analyzing the National Health Insurance Research Databases. Laboratory-confirmed dengue patients from 2002 to 2012 and four matched nondengue controls were identified. Multivariate Cox proportional hazard regression was used to evaluate the acute (<30 days), medium-term (31–365 days), and long-term (>365 days) risks of nonvariceal upper GI bleeding after DENV infection. Stratified analyses by age group (≤50, 51–64, ≥65 years old) were also performed. In total, 13267 confirmed dengue patients and 53068 nondengue matched controls were included. After adjusting for sex, age, area of residence, comorbidities, and medications, dengue patients had a significantly increased risk of nonvariceal upper GI bleeding within 30 days of disease onset (adjusted HR 55.40; 95% CI: 32.17–95.42). However, DENV infection was not associated with increased medium-term and long-term risks of upper GI bleeding overall or in each age group. Even dengue patients who developed acute GI bleeding did not have increased medium-term (adjusted HR; 0.55, 95% CI 0.05–6.18) and long-term risks of upper GI bleeding (adjusted HR; 1.78, 95% CI 0.89–3.55). DENV infection was associated with a significantly increased risk of nonvariceal upper GI bleeding within 30 days but not thereafter. Recovered dengue patients with acute GI bleeding can resume antithrombotic treatments to minimize the risk of thrombosis.

## Author summary

Dengue fever is a mosquito-borne tropical disease caused by the dengue virus. Dengue patients can have low platelet counts and might have acute gastrointestinal bleeding (tarry stool, bloody stool or bloody vomiting). Most dengue patients will fully recover and return to their previous health levels. Previous studies have indicated that some dengue patients have persistent low platelet counts and high inflammatory responses. The medium-term and long-term upper gastrointestinal bleeding risks remain unknown. Our study suggested that dengue was significantly associated with an increased risk of nonvariceal upper GI bleeding within 30 days after infection but was not associated with increased medium-term (31–365 days) and long-term risks (>365 days) of upper GI bleeding. Therefore, the risk of acute gastroenterology bleeding returned to baseline levels after 30 days. Recovered dengue patients with acute GI bleeding can resume antiplatelet, antithrombotic, and oral anticoagulation (OAC) treatments.

## Introduction

Dengue fever, caused by dengue virus (DENV) infection, has become a significant global public health challenge due to its dramatically increasing incidence over 50-fold in the past five decades and continuing geographical expansion to new regions [1,2]. DENV infection manifests as a spectrum of clinical severity that includes asymptomatic infection, classic dengue fever (DF), and severe dengue, previously known as dengue hemorrhagic fever/dengue shock syndrome (DHF/DSS) [3]. Expanded dengue syndrome was coined by the World Health Organization (WHO) in 2012 to describe cases that did not fall into either DHF or DSS. Unusual manifestations with severe organ involvement, such as liver, kidneys, brain or heart involvement, have been increasingly reported in DHF cases and dengue patients without evidence of plasma leakage. These unusual manifestations may be associated with coinfections, comorbidities or complications of prolonged shock [4–6]. Bleeding manifestations, one of the major characteristics of dengue, can range from epistaxis, gingival bleeding, and substantial menstruation to gastrointestinal (GI) bleeding [7]. GI bleeding has been shown to be an indicator of poor prognosis in dengue patients and requires complex intensive supportive care [8,9].

Dengue has generally been viewed as an acute infection without long-term consequences. However, increasing evidence suggests that DENV infection may have long-term health effects such as persistent dengue-related symptoms, altered autoimmune titers, and an increased risk of developing leukemia at more than three years after infection [10–12]. GI bleeding is frequently observed in severe dengue and dengue patients with thrombocytopenia. However, the duration of increased upper GI bleeding risk and the long-term risk of upper GI bleeding remain unknown. Recently, the changing epidemiological trends of dengue have resulted in an increasing number of adult and elderly dengue patients [13–21]; some of them may take antiplatelet and anticoagulant therapy regularly to prevent cardiovascular events, and treatment interruption may increase the risk of thrombosis. On the other hand, antithrombotic drugs will increase the risk of upper GI bleeding, and thus temporary discontinuation of the drugs during massive GI bleeding is recommended [22]. However, the time to resume antiplatelets and anticoagulants in dengue patients to balance the risks of thrombosis and GI bleeding is still uncertain.

Large-scale cohort studies can be very costly to conduct and are affected by bias resulting from loss to follow-up. The comprehensiveness and availability of nationwide health databases in Taiwan offer a valuable opportunity to evaluate the potential long-term health effects of DENV infection. Therefore, this study adopted national databases to assess the short-term, medium-term, and long-term risks of upper GI bleeding among dengue patients to guide the clinical care of dengue.

## Methods

### Ethics statement

This study was reviewed and approved by the Institutional Review Board of National Cheng Kung University Hospital (B-ER-106-184). The national databases used in this study were anonymized and deidentified by the Health and Welfare Data Science Center (HWDC) in Taiwan, and thus informed consent was waived. Under the regulation of HWDC, the data must be accessed and analyzed in a restricted area, so the data cannot be shared. In addition, case numbers fewer than three were not allowed to be reported in the analyses to prevent reidentification.

### Data sources

This retrospective cohort study was conducted using the Health and Welfare Database established by the Health and Welfare Data Science Center under the supervision of the Ministry of Health and Welfare of Taiwan. Among the various data sources included in the database, detailed claims data from the National Health Insurance (NHI) program, which has enrolled more than 99% of over 23 million residents in Taiwan [23], have become essential for healthcare-related research in recent years. All individual information stored in the database is well protected via encrypted personal identification numbers, which allows mutual linkage to different national databases, such as the Cause of Death Database, Cancer Registry, and the Notifiable Disease Dataset of Confirmed Cases. According to the Communicable Disease Control Act in Taiwan, dengue fever is classified as a category 2 notifiable communicable disease that should be reported to government health authorities within 24 hours. Blood specimens from suspected cases were tested by laboratories certified by the Taiwan Centers for Disease Control to confirm the diagnosis. The criteria for laboratory confirmation during the study period from 2002 to 2012 included any of the following: isolation of DENV, positive results using real-time reverse transcription polymerase chain reaction, a fourfold rise in the IgG titer in paired acute- and convalescent-phase samples, or detection of dengue-specific IgM and IgG antibodies in a single serum sample [24].

### Study population with inclusion and exclusion criteria

**Fig 1:**
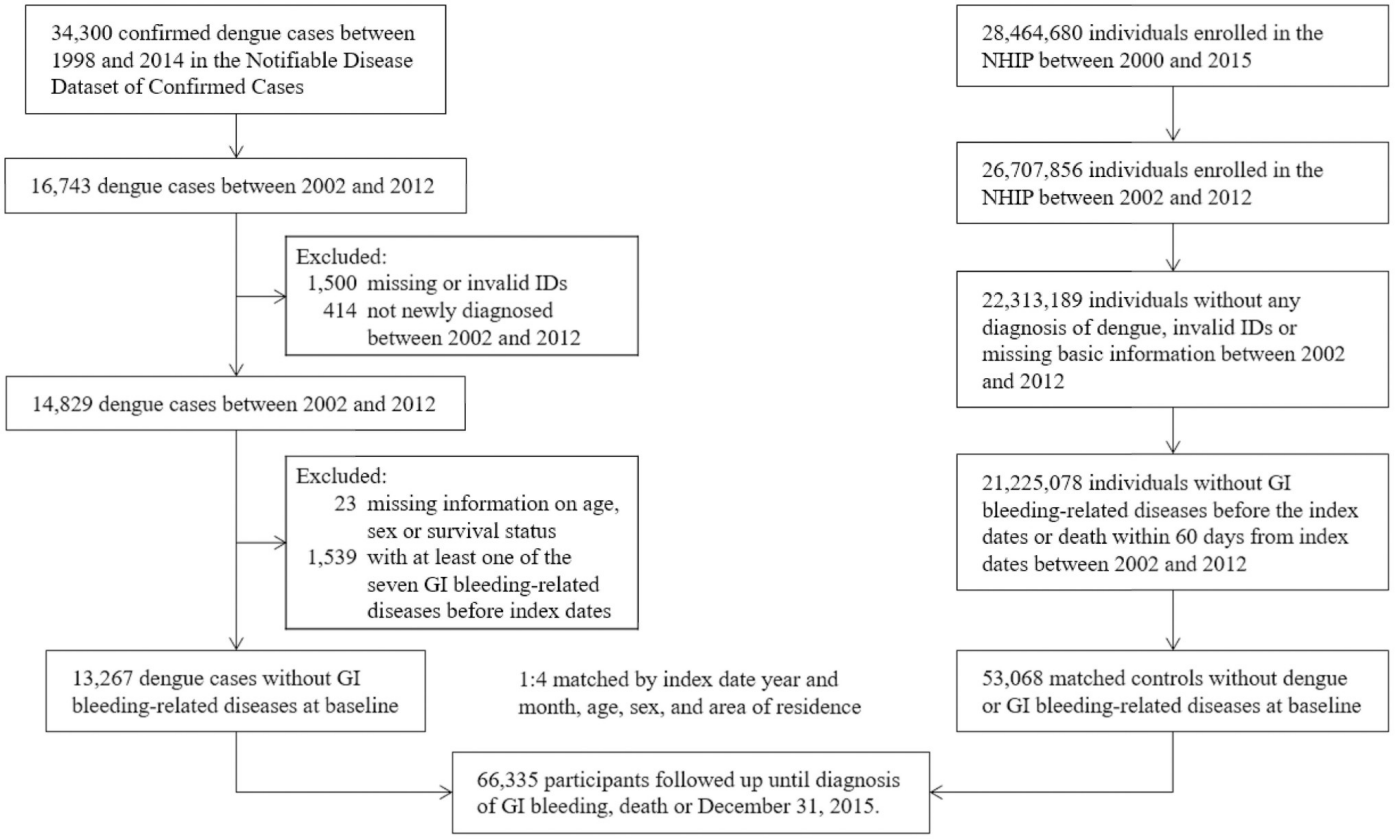
Flow diagram of the selection of the study population.

Newly confirmed dengue cases from 2002 to 2012 were identified from the Notifiable Disease Dataset of Confirmed Cases; those without valid identification numbers or not enrolled in the NHI program were excluded (Fig 1). The date of symptom onset for each dengue case was defined as the index date. Individual matching was performed to randomly match four nondengue controls to each confirmed dengue patient by age, sex, area of residence (Tainan, Kaohsiung, Pingtung, and others), and the calendar year of the index date; the index dates for the matched controls were the same as those for their corresponding dengue patients. Those in both the dengue and nondengue groups with alcohol-related diseases, GI tract malignancies, coagulopathy, vascular insufficiency of the intestine, gastroenteritis or colitis due to radiation, or any GI hemorrhage before the index date were excluded. These conditions were defined as at least two outpatient visits or one hospital admission with relevant International Classification of Diseases, Ninth Revision, Clinical Modification (ICD-9-CM) codes (S1 Table). The death dates for study participants were retrieved from the Cause of Death Database.

### Study outcome and follow-up

As the main outcome of interest in this study, nonvariceal upper GI bleeding was defined by one hospital admission with the following ICD-9-CM codes: 530.21, 530.7, 530.82, 531.0, 531.2, 531.4, 531.6, 532.0, 532.2, 532.4, 532.6, 533.0, 533.2, 533.4, 533.6, 534.0, 534.2, 534.4, 534.6, 535.01, 535.11, 535.21, 535.31, 535.41, 535.51, 535.61, 535.71, 537.83, 537.84. It is well known that acute dengue infection can cause GI bleeding; thus, we first calculated the incidence of nonvariceal upper GI bleeding within 30 days after the index date in the dengue and nondengue groups. Since the increased risk of upper GI bleeding in acute DENV infection is well known, the main study objective was to investigate the medium-term (31–365 days after symptom onset) and long-term risks of upper GI bleeding (>365 days after symptom onset). Therefore, we followed the participants from 31 days after the index date to 1) the occurrence of the study outcome; 2) death without a diagnosis of GI bleeding; or 3) December 31, 2015, whichever occurred first. Participants who had nonvariceal upper GI bleeding within 30 days after the index date were not excluded and were followed after 30 days to investigate the medium-term and long-term risks of nonvariceal upper GI bleeding.

### Definition of covariates

In addition to age and sex, area of residence was incorporated into the research design as a demographic variable because most dengue cases were geographically located in southern Taiwan, including Tainan, Kaohsiung, and Pingtung, where *Aedes aegypti* is prevalent, while there were only sporadic cases or small clusters in other parts of Taiwan. Comorbidities considered in this study included hypertension, diabetes mellitus (DM), coronary artery disease, chronic obstructive pulmonary disease (COPD), liver cirrhosis, uncomplicated peptic ulcer disease, dyslipidemia, and ischemic stroke, which were defined by at least three outpatient visits or one hospital admission before the index date with relevant ICD-9-CM codes (S1 Table). Medications including acetylsalicylic acid, thienopyridine and dipyridamole, nonsteroidal anti-inflammatory drugs (NSAIDs), steroids, anticoagulants, and selective serotonin reuptake inhibitors (SSRIs) that had been prescribed within six months before the index date were retrieved from the NHI claims database and analyzed as potential confounders (S2 Table). The covariates listed above were measured using the same methods from the NHIRD for the dengue and nondengue groups.

### Statistical analysis

Chi-square tests were used to compare the baseline characteristics between the dengue and nondengue groups. Since the sample size of this study was large, the differences might be statistically significant but not clinically meaningful using traditional significance tests. Therefore, the standardized differences (SDs) were also calculated to compare differences, and an SD greater than 0.1 was regarded as a meaningful difference [25]. For both groups, the incidence rate of nonvariceal upper GI bleeding was calculated as the number of events during the follow-up period divided by the total follow-up time in person-months. Univariate and multivariate Cox proportional hazards regression models were used to estimate hazard ratios (HRs) and 95% confidence intervals (CIs) for nonvariceal upper GI bleeding in dengue patients compared to nondengue controls, controlling for the abovementioned demographic variables, comorbidities, and medications. We performed stratified analyses by time and calculated the HRs of GI bleeding at different follow-up times (≤ 30 days and > 30 days after the index date); follow-up times > 30 days were further split into 31–365 days and > 365 days to investigate the medium-term and long-term risks of upper GI bleeding after DENV infection. Analyses stratified by age were also performed to investigate the effect of age on the association between DENV infection and GI bleeding. In addition, we examined whether dengue patients with acute GI bleeding in the first 30 days had an increased risk of upper GI bleeding after 30 days. Furthermore, because adults and elderly individuals accounted for the majority of participants in the study and the follow-up time was quite long, subdistribution HRs were also calculated using the Fine and Gray model to address the possible competing risk of mortality in sensitivity analyses [26]. All data were analyzed with SAS 9·4 (SAS Institute, Cary NC), and the level of statistical significance in this study was set at 0.05 by convention.

## Results

The selection of patients and controls is illustrated in Fig 1 (flow diagram of the selection of the study population). A total of 13267 confirmed dengue patients eligible for this study and 53068 nondengue matched controls were included in this study. The median follow-up times were 8.11 years (IQR 5.07–13.08) and 8.13 years (IQR 5.11–13.11) in the dengue and nondengue groups, respectively. The baseline demographic characteristics, comorbidities, and medications prescribed within six months before the index date are listed in Table 1. Although the dengue group seemed to have a higher prevalence of several comorbidities than the nondengue group using Chi-square tests, there was no meaningful difference in the prevalence of any of the selected comorbidities between the two groups using SDs. The use of NSAIDs before the index date was higher in the dengue group than in the nondengue group.

**Table 1:** Demographic and clinical characteristics in the dengue and nondengue groups.

	Dengue cohort (N = 13267)	Nondengue cohort (N = 53068)	χ^2^ p-value	Standardized difference
Sex				
Female	6701 (50.5)	26804 (50.5)		-
Male	6566 (49.5)	26264 (49.5)		-
Age (years)	44.4 (18.6) *	44.4 (18.6) *		-
0–17	1279 (9.7)	5116 (9.7)		-
18–35	3055 (23.0)	12220 (23.0)		-
36–50	3436 (25.9)	13744 (25.9)		-
51–64	3616 (27.3)	14464 (27.3)		-
≥65	1881 (14.2)	7524 (14.2)		-
Area of residence				
Tainan	2875 (21.7)	11500 (21.7)		-
Kaohsiung	8668 (65.3)	34672 (65.3)		-
Pingtung	642 (4.8)	2568 (4.8)		-
Others	1082 (8.2)	4328 (8.2)		-
Comorbidity				
Hypertension	2759 (20.8)	9778 (18.4)	<.0001	0.061
Diabetes mellitus	1351 (10.2)	4687 (8.8)	<.0001	0.047
Coronary artery disease	1136 (8.6)	3618 (6.8)	<.0001	0.067
COPD	732 (5.5)	2747 (5.2)	0.115	0.015
Chronic renal disease	334 (2.5)	1207 (2.3)	0.096	0.016
Liver cirrhosis	65 (0.5)	279 (0.5)	0.608	0.005
Uncomplicated PUD	1341 (10.1)	4681 (8.8)	<.0001	0.045
Dyslipidemia	1704 (12.8)	5514 (10.4)	<.0001	0.079
Ischemic stroke	280 (2.1)	1128 (2 1)	0.914	0.001
Medication				
Acetylsalicylic acid	829 (6.3)	2598 (4.9)	<.0001	0.061
NSAIDs	7385 (55.7)	24162 (45.5)	<.0001	0.204
Steroids	2010 (15.2)	6804 (12.8)	<.0001	0.069
Thienopyridine	96 (0.7)	284 (0.5)	0.010	0.025
Dipyridamole	365 (2.8)	1135 (2.1)	<.0001	0.041
Anticoagulants	38 (0.3)	140 (0.3)	0.653	0.004
SSRIs	142 (1.1)	534 (1.0)	0.511	0.006

Data are expressed in number and percentage, except for the variable “age,” of which the *mean and standard deviation are also shown.

COPD = chronic obstructive pulmonary disease.

NSAIDs = nonsteroidal anti-inflammatory drugs

SSRIs = selective serotonin reuptake inhibitors

PUD = peptie ulcer disease

Among the 13267 confirmed dengue patients, 195 (1.47%) patients developed nonvariceal upper GI bleeding within 30 days after symptom onset, and 247 (1.86%) had events after 30 days, while among the 53068 non-dengue controls, 14 (0.03%) and 939 (1.77%) individuals develop GI bleeding within 30 days and after 30 days, respectively. The incidence rates of nonvariceal upper GI bleeding in the dengue and nondengue groups were 15.14 and 0.27 per 1000 person-months within 30 days after symptom onset and 0.19 and 0.18 per 1000 person-months after 30 days, respectively (Table 2). After adjusting for sex, age, area of residence, comorbidities, and medications listed in Table 1, DENV infection significantly increased the risk of nonvariceal upper GI bleeding within 30 days after the index date (adjusted HR 55.40; 95% CI: 32.17–95.42; P < 0.0001; Table 2). However, DENV infection was not associated with an increased risk of upper GI bleeding more than 30 days after the index date (adjusted HR 0.97; 95% CI: 0.84–1.12; P = 0.692). Further stratified by follow-up time, the results showed that people with previous DENV infection did not have a higher medium-term (31–365 days, adjusted HR 0.80; 95% CI: 0.51–1.26; P = 0.338) or long-term risk (>365 days, adjusted HR 0.99; 95% CI: 0.86–1.15; P = 0.936) of upper GI bleeding after DENV infection. Sensitivity analysis showed that the subdistribution HRs obtained from the Fine and Gray models were similar to the HRs from the Cox proportional hazards regression models (Table 2).

**Table 2:** Comparison of the incidence of nonvariceal upper GI bleeding.

Day	Dengue cohort		Nondengue cohort		Crude HR (95% CI)	P-value	Adjusted HR* (95% CI)	P-value	Adjusted SHR† (95% CI)	P-value
	No. of events	Incidence rate (per 1000 person-months)	No. of events	Incidence rate (per 1000 person-months)						
≤30	195	15.14	14	0.27	56.12 (32.63–96.52)	< 0.0001	55.40 (32.17–95.42)	< 0.0001	55.34 (32.10–95.44)	< 0.0001
> 30	247	0.19	939	0.18	1.05 (0.91–1.21)	0.485	0.97 (0.84–1.12)	0.692	1.00 (0.87–1.16)	0.956
31–365	23	0.16	110	0.19	0.84 (0.54–1.32)	0.444	0.80 (0.51–1.26)	0.338	0.81 (0.51–1.27)	0.353
> 365	224	0.19	829	0.18	1.08 (0.93–1.25)	0.319	0.99 (0.86–1.15)	0.936	1.02 (0.88–1.19)	0.797

Adjusted HR*: Hazard ratio adjusted for age, sex, area of residence, comorbidities, and medications listed in Table 1.

Adjusted SHR† Subdistribution hazard ratio adjusted for age, sex, area of residence, comorbidities, and medications listed in Table 1.

Considering age effects on the risk of GI bleeding, we also stratified the data by age and reanalyzed the incidence of upper GI bleeding in the dengue and nondengue groups (Table 3). During the acute phase (within 30 days) after dengue infection, the incidence of nonvariceal GI bleeding increased with age (≤50 years: 8.82 per 1000 person-months; 51–64 years: 20.62 per 1000 person-months; ≥65 years: 31.27 per 1000 person-months); however, the HR of upper GI bleeding in the dengue cohort compared to the nondengue cohort was highest in the young group (≤50 years: adjusted HR 85.42; 95% CI, 26.83–271.93; P < 0.0001; 51–64 years: adjusted HR 73.68; 95% CI: 26.86–202.06; P < 0.0001; ≥65 years: adjusted HR 31.78; 95% CI, 14.43–70.01; P < 0.0001) (Table 3). Similar to the results of the previous analysis, the results showed that people with previous DENV infection did not have an increased medium-term (31–365 days) (≤50 years: adjusted HR 2.05; 95% CI: 0.66–6.43; P = 0.217; 51–64 years: adjusted HR: 0.86; 95% CI: 0.40–1.87; P = 0.707; ≥65 years: adjusted HR: 0.60; 95% CI: 0.31–1.17; P = 0.134) or long-term risk (>365 days) (≤50 years:, adjusted HR 0.77; 95% CI: 0.54–1.11; P = 0.168; 51–64 years: adjusted HR: 1.04; 95% CI: 0.81–1.32; P = 0.772; ≥65 years: adjusted HR 1.05; 95% CI: 0.85–1.31; P = 0.646) of upper GI bleeding after DENV infection in all age groups (Table 3).

**Table 3:** Comparison of the incidence of nonvariceal upper GI bleeding (stratified by age).

Age	Days after infection	Dengue cohort		Nondengue cohort		Crude HR (95% CI)	P-value	Adjusted HR* (95% CI)	P-value	Adjusted SHR† (95% CI)	P-value
		No. of events	Incidence rate (per 1000 person-months)	No. of events	Incidence rate (per 1000 person-months)						
≤ 50	≤ 30	67	8.82	3	0.10	89.64 (28.20–284.92)	< 0.0001	85.42 (26.83–271.93)	< 0.0001	85.41 (26.96–270.61)	< 0.0001
	> 30	41	0.05	178	0.06	0.92 (0.66–1.29)	0.630	0.85 (0.61–1.20)	0.360	0.87 (0.61–1.22)	0.409
	31–365	5	0.06	8	0.02	2.50 (0.82–7.65)	0.108	2.05 (0.66–6.43)	0.217	2.05 (0.68–6.12)	0.200
	> 365	36	0.05	170	0.06	0.85 (0.59–1.21)	0.360	0.77 (0.54–1.11)	0.168	0.79 (0.55–1.14)	0.199
51–64	≤ 30	72	20.62	4	0.28	72.72 (26.57–199.03)	< 0.0001	73.68 (26.86–202.06)	< 0.0001	73.59 (26.83–201.84)	< 0.0001
	> 30	92	0.25	332	0.23	1.11 (0.88–1.40)	0.386	1.02 (0.81–1.29)	0.865	1.04 (0.82–1.32)	0.725
	31–365	8	0.20	36	0.23	0.89 (0.42–1.92)	0.772	0.86 (0.40–1.87)	0.707	0.87 (0.40–1.88)	0.716
	> 365	84	0.26	296	0.23	1.13 (0.89–1.44)	0.313	1.04 (0.81–1.32)	0.772	1.06 (0.83–1.36)	0.657
≥ 65	≤ 30	56	31.27	7	0.94	32.53 (14.83–71.36)	< 0.0001	31.78 (14.43–70.01)	< 0.0001	31.72 (14.29–70.41)	< 0.0001
	> 30	114	0.71	429	0.67	1.06 (0.86–1.30)	0.596	0.98 (0.80–1.21)	0.881	1.02 (0.83–1.26)	0.829
	31–365	10	0.50	66	0.81	0.61 (0.32–1.19)	0.148	0.60 (0.31–1.17)	0.134	0.60 (0.31–1.19)	0.144
	> 365	104	0.73	363	0.64	1.14 (0.91–1.41)	0.248	1.05 (0.85–1.31)	0.646	1.09 (0.87–1.35)	0.454

Adjusted HR*: Hazard ratio adjusted for age, sex, area of residence, comorbidities, and medications listed in Table 1.

Adjusted SHR† Subdistribution hazard ratio adjusted for age, sex, area of residence, comorbidities, and medications listed in Table 1.

We further analyzed whether the 195 dengue patients who developed acute GI bleeding in the first 30 days had an increased risk of upper GI bleeding after 30 days. Compared to their matched nondengue controls, the dengue patients with GI bleeding during the acute phase did not have an increased medium-term (adjusted HR; 0.55, 95% CI 0.05–6.18, P = 0.631) or long-term risk of upper GI bleeding (adjusted HR; 1.78, 95% CI 0.89–3.55, P = 0.103).

## Discussion

Dengue is not considered endemic in Taiwan. Historically, there were several dengue outbreaks in Taiwan before and during World War II, including a severe outbreak in 1942–43, probably resulting from substantial migration and travel during the war [27–29]. After the war, no dengue patients were reported on the main island of Taiwan until 1987 due to the restriction on international travel under martial law. Since 1987, small dengue epidemics have been observed almost every year in southern Taiwan, mostly involving only a few hundred to more than one thousand patients before 2013 [27,28]. However, two severe dengue outbreaks occurred in 2014 and 2015, resulting in approximately 15,000 and 43,000 patients, respectively. Only small outbreaks or clusters occurred thereafter. In this study, dengue patients diagnosed between 2002 and 2012 were included, and thus, they were more likely to have primary dengue infection.

As expected, this study suggested a significantly increased incidence of nonvariceal upper GI bleeding in dengue patients within 30 days after acute DENV infection compared to the general population. However, even though the HR was very high, only 195 (1.47%) of 13267 dengue patients had acute upper GI bleeding, revealing that acute GI bleeding was uncommon in this population composed mainly of adults and elderly individuals with primary dengue infection. The risk of upper GI bleeding in dengue patients returned to baseline levels after 30 days of symptom onset.

Previous studies have reported that approximately 8–13% of dengue hospitalization patients had GI bleeding episodes [30–32]. Approximately 7% of pediatric dengue hospitalization patients have symptoms of GI bleeding, such as melena and hematemesis [33], and 40% of severe dengue patients have GI bleeding [34]. The reported incidence of GI bleeding in the literature seems to be much higher than that in our study, probably because previous studies were mainly hospital-based studies recruiting patients with higher severity, while ours was population-based. Multiple factors are attributed to GI bleeding in dengue patients, such as abnormalities in platelet function, thrombocytopenia, hyperfibrinolysis and reduced synthesis of coagulation factors as a result of associated hepatitis [6,35]. DENV infection is associated with altered production of inflammatory and vasoactive factors and functional changes in the endothelium and platelet function [36]. DENV nonstructural protein 1 (NS1) directly activates Toll-like receptor 4 (TLR4)-expressing immune cells to trigger the secretion of proinflammatory cytokines that cause endothelial dysfunction and thrombocytopenia [37,38]. NS1 may stimulate the secretion of other soluble molecules with vasoactive and proteolytic activities that can affect endothelial barrier integrity [37]. Cross-reactive anti-NS1 antibodies may bind to platelets and components of the clotting cascade (e.g., plasminogen and thrombin). In addition, thrombocytopenia is thought to result from early pancytopenic suppression of bone marrow, either by direct infection of megakaryocytes or by activated T-cell suppression of hematopoiesis [39,40]. Peripheral immune-mediated platelet destruction also occurs via DENV binding to platelets [41]. In addition to DENV-associated imbalance between clotting and fibrinolysis systems, non-DENV-associated factors, including advanced age, sepsis-associated stress ulcers, worsening of preexisting peptic ulcers, underlying comorbidities such as cirrhosis and end-stage renal diseases, and medications (aspirin, anti-coagulants, and NSAIDs) interfering with platelet or coagulating functions, also contributed to acute GI bleeding [30,32,42]. However, this study only utilized insurance claim data, and thus GI bleeding caused by different pathologies in dengue patients could not be discriminated because detailed clinical information and laboratory findings were not available.

Endoscopic findings of dengue-associated upper GI bleeding included hemorrhagic and/or erosive gastritis in 67% of patients, gastric ulcers in 57.7%, duodenal ulcers in 26.8%, and esophageal ulcers in 3.1% [30,32]. Choices of treatments for dengue patients with GI bleeding included bleeding tendency correction, proton pump inhibitor (PPI) infusion, and endoscopic injection. Although PPI infusion and endoscopic injection are gold standards in treating gastric and duodenal ulcers of upper GI bleeding, injection site bleeding has been observed in dengue patients who have thrombocytopenia and bleeding tendency; therefore, endoscopic injection therapy is not recommended [32]. Prophylactic platelet transfusions to prevent bleeding have not been shown to be effective in dengue infection; however, blood transfusion is lifesaving and should be given as soon as severe bleeding is recognized [43].

Adult and elderly patients with comorbidities have increased risks of severe dengue and mortality [44]. These patients frequently take antiplatelet and oral anticoagulation (OAC) agents to prevent thrombus formation. NSAIDs and steroids are also common treatments for arthritis (rheumatoid arthritis, osteoarthritis, and others) and lupus. Studies have suggested that patients with moderate to severe nonvariceal upper GI bleeding should temporarily discontinue the use of antiplatelet, antithrombotic, and OAC agents to decrease the risk of uncontrolled bleeding; resuming these medications to reduce the risk of thrombosis and death should be considered after bleeding resolves [22]. The timeframe for the resumption of therapy ranged from 20 to 90 days after GI bleeding had stopped [45]. For dengue patients with a low short-term risk, such as patients with stable coronary artery disease, temporarily interrupting the use of antithrombotic agents is usually recommended [46]. There is currently no clear guideline regarding the best times to withhold and resume antithrombotic agents in dengue patients. In most recovered dengue patients, platelet function is restored to the preinfection level. Although elderly individuals and dengue patients with comorbidities have higher risks of progression to severe dengue and nonvariceal upper GI bleeding, they also require long-term medications to reduce the risk of thrombosis. Our study found that the risk of nonvariceal upper GI bleeding in the dengue group was similar to that in the nondengue group at more than 30 days after acute dengue infection in all age groups, even in dengue patients with acute GI bleeding. Therefore, resuming antiplatelet and OAC agents to reduce the risk of cardiovascular events for acute DENV infection patients after 30 days of upper GI bleeding should be safe. However, whether these drugs should be resumed earlier to balance the risk of thrombosis and bleeding requires further study to aid in the development of evidence-based guidelines for the treatment of dengue patients.

In dengue hyperendemic countries where multiple serotypes cocirculate for a long time, the majority of dengue patients are children because adults and elderly individuals have been previously exposed to the virus and have immunity [21,47]. However, a shift toward older age groups in patients with dengue fever or dengue hemorrhagic fever has been observed in many hyperendemic countries, such as Singapore [17], Thailand [13–15], Indonesia [18], Malaysia [19], and India [48]. This age shift in dengue cases may be a consequence of vector control programs causing the decreasing force of infection and lower herd immunity in adults as well as the demographic transition due to decreasing birth rates and increasing life expectancy [14,17,49]. Over the past few decades, dengue has spread to new regions and countries where dengue transmission activity remains low [50]. Although people in all age groups are equally susceptible to dengue in these nonendemic areas, the current population age structure should cause more cases in adults and elderly individuals than in children, as seen in Taiwan. As a result, the dengue disease burden in older adults and elderly individuals may be continuing to increase. However, the management of dengue in these age groups remains understudied [16]. Our data could help to guide dengue treatment among older adults and elderly individuals, especially in nonendemic countries, where most people should have primary DENV infections and different immunological profiles from people in endemic or hyperendemic countries.

Our study has several strengths. First, our study was a large-scale, population-based, long-term follow-up study. We adapted a well-designed cohort study by using national notifiable infectious disease report data and medical claims data to evaluate the clinical course and long-term health consequences in dengue patients. This large-scale population-based study minimized the effects of selection bias, and the high coverage of the NHI program minimized loss to follow-up. Second, all dengue cases were laboratory confirmed. Third, we controlled for many factors, including sex, age, demographics, socioeconomic factors, comorbidities, and medications, to eliminate potential confounding effects that might affect the risk of upper GI bleeding. Fourth, the risk of nonvariceal upper GI bleeding was assessed in different age groups. No noticeable difference in the long-term risk of nonvariceal upper GI bleeding was observed in elderly dengue patients who might have taken antiplatelets and anticoagulants. Finally, we also performed sensitivity analyses using subdistribution hazard models to account for potential competing risks, and the results of the sensitivity analyses were very similar to those in the original analysis.

Several limitations in this study need to be mentioned. First, the DENV infection status in some people in the nondengue group might have been misclassified because not all DENV-infected individuals seek medical care, and DENV cases can be missed by the surveillance system because the majority of DENV infections produce no symptoms or are very mild. However, most DENV infections in Taiwan occur in adults, who have been reported to be more likely to develop classic dengue fever than children [47,51–53] and thus might be more likely to be recognized in the surveillance system. In addition, although several dengue epidemics have occurred in Taiwan, dengue is not endemic in Taiwan, and the overall seroprevalence of DENV infection remains very low in most parts of Taiwan [54]. Accordingly, this misclassification bias should be very low. Second, the databases we used lacked information on potential confounders, such as smoking status and alcohol consumption, which are also risk factors for upper GI bleeding. Third, this study investigated only the risk of nonvariceal upper GI bleeding; we attempted to explore the risk of lower GI bleeding after DENV infection, but the case number was too low after stratification by follow-up time; thus, the results were not reported. Under the regulation of the Health and Welfare Data Science Center in Taiwan, data from fewer than three individuals cannot be exported; this prevents reidentification and protects individual privacy. Fourth, among dengue patients with acute upper GI bleeding, we only knew that they had been hospitalized within 30 days after the onset of dengue symptoms and had a discharge diagnosis of upper GI bleeding; however, the exact dates on which GI bleeding occurred were not known. This was because the databases we used were claims data used for insurance reimbursements rather than the detailed medical records. Therefore, it was difficult to stratify the patients based on the duration for further analysis. Finally, we could not differentiate whether the dengue patients had a primary or secondary infection because the characterization of types of antibody response is not routinely performed in Taiwan. However, the dengue patients included in this study were infected between 2002 and 2012, the time before the severe epidemics in 2014 and 2015, and thus were more likely to have primary infections.

In conclusion, we found a significantly increased risk of nonvariceal upper GI bleeding among dengue patients within 30 days after acute dengue infection, as expected. However, the increased risk of upper GI bleeding was not sustained at more than 30 days after acute dengue infection in all age groups, even among dengue patients with acute GI bleeding. Recovered dengue patients with acute GI bleeding can resume the use of antiplatelet, antithrombotic, and OAC agents to minimize the risk for thrombosis after 30 days.

## Supporting information

S1 Table List of ICD-9-CM codes for identifying GI bleeding-related diseases and comorbidities. (DOCX)

S2 Table Enrolled Drugs and Anatomical Therapeutic Chemical (ATC) codes. (DOCX)
