# Supplementary material for: Short-term, medium-term, and long-term risks of nonvariceal upper gastrointestinal bleeding after dengue virus infection
Source: PLoS Negl Trop Dis. 2022 Jan 19;16(1):e0010039. doi: 10.1371/journal.pntd.0010039 (PMC8769317; doi:10.1371/journal.pntd.0010039)
Supplement: S1 Table — (DOCX) [file pntd.0010039.s001.docx]

### S1 Table. List of ICD-9-CM codes for identifying GI bleeding-related diseases and comorbidities

| **GI bleeding and related diseases** | **ICD-9-CM** |
| --- | --- |
| Alcohol-related disease | 291, 303, 305, 571.0, 571.1, 571.2, 571.3 |
| Malignancy of the GI tract | 150, 151, 152, 153, 154 |
| Inflammatory bowel disease | 555, 556 |
| Coagulopathy | 286 |
| Vascular insufficiency of the intestine | 557 |
| Gastroenteritis or colitis due to radiation | 558.1 |
| GI bleeding/hemorrhage |  |
| Nonvariceal upper GI bleeding | 530.21, 530.7, 530.82, 531.0, 531.2, 531.4, 531.6, 532.0, 532.2, 532.4, 532.6, 533.0, 533.2, 533.4, 533.6, 534.0, 534.2, 534.4, 534.6, 535.01, 535.11, 535.21, 535.31, 535.41, 535.51, 535.61, 535.71, 537.83, 537.84 |
| Lower GI bleeding | 569.85, 569.86, 562.02, 562.03, 562.12, 562.13, 569.3 |
| GI bleeding, none of the above | 456.0, 456.20, 578 |
| **Comorbidities** |  |
| Hypertension | 401-405 |
| Diabetes mellitus | 250 |
| Coronary artery disease | 411-414 |
| COPD | 491, 492, 494, 496 |
| Chronic renal disease | 585, 586, 588.8,588.9, 250.4, 274.1, 403.x1, 404.x2, 404.x3, 440.1 |
| Liver cirrhosis | 571.2, 571.5, 571.6 |
| Uncomplicated peptic ulcer disease | 531.30, 531.70, 531.90, 532.30, 532.70, 532.90, 533.30, 533.70, 533.90 |
| Dyslipidemia | 272.0, 272.1, 272.2, 272.3, 272.4 |
| Ischemic stroke | 433, 434 |
