# Supplementary material for: Short-term, medium-term, and long-term risks of nonvariceal upper gastrointestinal bleeding after dengue virus infection
Source: PLoS Negl Trop Dis. 2022 Jan 19;16(1):e0010039. doi: 10.1371/journal.pntd.0010039 (PMC8769317; doi:10.1371/journal.pntd.0010039)
Supplement: S2 Table — (DOCX) [file pntd.0010039.s002.docx]

**S2 Table Enrolled Drugs and Anatomical Therapeutic Chemical (ATC) codes**

| **Drugs** | **ATC code** |
| --- | --- |
| Acetylsalicylic acid (oral) | B01AC06 |
| Nonsteroidal anti-inflammatory drugs (NSAIDs) (oral or parenteral) | M01AB16, M01AA01, M01AB01, M01AB02, M01AB03, M01AB05, M01AB06, M01AB08,M01AB11, M01AB15, M01AB16, M01AC01, M01AC02,M01AC06, M01AE01,M01AE02, M01AE03, M01AE04, M01AE05, M01AE09, M01AE11, M01AE16, M01AE52, M01AG01, M01AG02, M01AG03, M01AG04, M01AH01, M01AH02, M01AH05, M01AX01, M01AX02, M01AX05, M01AX07, M01AX17, M01AX25, M01AX91, M01BA03 |
| Steroids (oral or parenteral) | H02AA02, H02AB01, H02AB02, H02AB04, H02AB06, H02AB08, H02AB09, H02AB10, H02BX, H02BX91 |
| Thienopyridine | B01AC04, B01AC05, B01AC22 |
| Dipyridamole | B01AC07 |
| Anticoagulants | B01AA03, B01AE07, B01AE07, B01AF01, B01AF02, B01AF03 |
| Selective serotonin reuptake inhibitors (SSRIs) | N06AB10, N06AB03, N06AB04, N06AB05, N06AB08 |
